# Rate of cesarean section among breech deliveries in Ethiopia: a systematic review and meta-analysis

**DOI:** 10.3389/fsurg.2024.1283965

**Published:** 2025-01-17

**Authors:** Ibsa Mussa, Adera Debella, Lemma Demissie Regassa, Badhasa Ahamed, Usmael Jibro, Addis Eyeberu

**Affiliations:** ^1^ School of Public Health, College of Health and Medical Sciences, Haramaya University, Harar, Ethiopia; ^2^ School of Nursing and Midwifery, College of Health and Medical Sciences, Haramaya University, Harar, Ethiopia; ^3^ Haramaya University Hiwot Fana Comprehensive Specialized Hospital, Harar, Ethiopia

**Keywords:** cesarean section—methods, breech delivery, systematic review, women, Ethiopia

## Abstract

**Background:**

Breech deliveries are a significant public health concern in developing countries. The World Health Organization (WHO) declared that the cesarean section rate should not be higher than 10%–15%. As unnecessary C-sections may be associated with an increased risk of maternal and neonatal mortality, this meta-analysis was aimed at determining the rate of caesarean sections among breech deliveries in Ethiopia.

**Methods:**

All published and unpublished articles were obtained from legitimate databases and websites. The PRISMA guidelines were used to conduct this systematic review and meta-analysis. The meta-analysis of the primary and secondary outcomes was performed using STATA version 18. The overall effect size with a 95% CI was estimated using the random effect model with the Der Simonian Liard method. A sensitivity analysis using a leave-one-out meta-analysis was computed.

**Results:**

This meta-analysis included a total of 57,236 mothers who had breech deliveries. The pooled prevalence of breech deliveries among women in Ethiopia was 5% [95% CI: 4, 6]. The overall pooled cesarean section rate among breech deliveries in Ethiopia was 41% (95% CI: 29–54).

**Conclusions:**

In this review, the pooled prevalence of breech deliveries among women in Ethiopia was 5%, and the overall rate of caesarian section among the breech deliveries was 41%. This finding pointed out that two out of every five pregnant women with breech presentation gave birth by cesarean section in Ethiopia. Therefore, the finding implies that both the government and all the concerned stakeholders shall be given particular emphasis made on strengthening antenatal care services and ensure more women have access to skilled healthcare professionals during childbirth. This can help in providing appropriate interventions, support to women and reducing the need for emergency and unnecessary breech deliveries. The result of this research are a baseline data for future researchers to conduct further studies to better understand the reasons behind the high rates and identify potential interventions and solutions specific to the African context.

## 1 Introduction

Breech presentation is the most common form of malpresentation, accounting for 3%–4% of all deliveries at term (1). Persistent breech presentation may be associated with abnormalities of the baby, the amniotic fluid volume, the placental localization, or the uterus. It may be due to an otherwise insignificant factor, such as corneal placental position, or it may be due to chance (2). Many breech births’ resulted in very high perinatal mortality and morbidity due to a combination of trauma, birth asphyxia, prematurity, and malformation. One study in Ethiopia reported that 19.4% of neonates undergoing term breech deliveries have long-term morbidity up to school age, irrespective of the mode of delivery (3).

A wide range of management policies have been instituted to reduce perinatal morbidity and mortality. On the other hand, the breech presentation delivery approach is a controversial issue in obstetrics. How to cope with breech delivery (vaginal or C-section) has been discussed to find the safest in terms of morbidity (4). However, a planned caesarean section is better than a planned vaginal birth for the term fetus in the breech presentation (5, 6). The Term Breech Trial study revealed that perinatal mortality or serious neonatal morbidity was significantly lower for the planned caesarean section group than for the planned vaginal birth group (7).

The American College of Obstetricians and Gynecologists recommends that cesarean delivery be offered to women with a breech presentation if certain criteria are met, such as if the estimated fetal weight is greater than 3,500 g, if the mother has a contracted pelvis or other factors that may make vaginal delivery difficult, or if the baby is in an incomplete breech presentation (i.e., one or both feet are below the baby's buttocks) (8). Cesarean delivery may also be recommended if the baby has a known medical condition that may make vaginal delivery risky. It is important to discuss the risks and benefits of both vaginal and cesarean delivery with your healthcare provider to make an informed decision. Cesarean deliveries are usually performed to reduce the risks to the infant, such as when the fetus is in a breech position rather than headfirst in the birth canal (9). But the risks to the mother caused by the surgical procedure may be greater than with a normal vaginal delivery (10).

The benefits of cesarean section breech delivery include a lower risk of injury to the baby during delivery, a reduced risk of oxygen deprivation, and a lower risk of incontinence and sexual dysfunction after the birth of the baby (11, 6). However, this procedure also carries some risks, such as a higher risk of bleeding, infection, and complications with anesthesia (12). In comparison to vaginal delivery, cesarean-section breech delivery may have a longer recovery time for the mother and may increase the risk of future pregnancies requiring cesarean delivery. Additionally, vaginal delivery is associated with a lower risk of complications such as bleeding, infection, and blood clots (13).

The World Health Organization (WHO) declared that the cesarean section rate should not exceed 10%–15%. However, unnecessary C-sections may be associated with an increased risk of maternal and neonatal mortality (14, 15). Studies done in Ethiopia show that a wide range of rates of cesarean sections among breech deliveries have been measured in several regions. Of all studies, more than half (6) were conducted in the Oromia region (16–20), one study in Harari (16), one study in Amhara (17), one study in the southern nation and nationality region (18), and one study in Addis Ababa (19). A total of 57,236 mothers who gave breech deliveries at the health institution were included in this systematic review and meta-analysis. A summary of the main characteristics of the papers included in this systematic review and meta-analysis. Despite several studies on the rate of cesarean section among breech deliveries, no systematic meta-analysis or meta-regression studies on the Ethiopian scale have been performed. Hence, the current study was devoted to performing a systematic review, meta-analysis, and meta-regression regarding the rate of cesarean section among breech deliveries in Ethiopia.

## 2 Methods

### 2.1 Protocol and registration

This review was conducted to identify the prevalence of perinatal outcomes among breech delivery women in Ethiopia accordance to the Preferred Reporting Items for Systematic Reviews and Meta-Analyses (PRISMA) 2020 guideline (Supplementary Material S2).

### 2.2 Eligibility criteria

Studies that scrutinized the rate of cesarean sections in breech delivery among women in Ethiopia were included. The study included all observational studies that had either the primary or secondary outcomes of the review. The pre-specified criteria for inclusion were: Population: delivered women or postpartum women; study area: only studies conducted in Ethiopia; publication condition: both published and unpublished research; study design: all observational study designs; and language: studies reported in the English language were included. Finally, all full-text papers published up until August 13, 2023, were included. This review excluded case series or reports, reviews, commentaries, and editorials.

### 2.3 Information sources

The articles were searched and retrieved from valid and reliable databases and website platforms such as the Web of Sciences, MEDLINE, EMBASE, SCOPUS, PubMed, and Google Scholar. We also tried to access different universities’ institutional repository sites found in Ethiopia. A direct Google search was also performed.

### 2.4 Search strategy

The search was accomplished using Medical Subject Headings (MeSH) and keywords with a combination of Boolean logic operators (AND, OR, NOT). The search strategy for advanced PubMed includes “cesarean section” [MeSH Terms] AND ((“breech”[All Fields] OR “breeches”[All Fields]) AND (“deliveries”[All Fields] OR “delivery, obstetric”[MeSH Terms] OR (“delivery”[All Fields] AND “obstetric”[All Fields]) OR “obstetric delivery”[All Fields] OR “delivery”[All Fields])) AND “ethiopia”[MeSH Terms]. The search strategies are presented in Supplementary Material S1.

### 2.5 Study selection

The database search results were consolidated, and duplicate articles were manually removed using the reference management application (Endnote version X8). The titles and abstracts of the papers were then carefully evaluated. Three writers (IM, LDR, and AE) independently reviewed the full texts of the remaining publications to determine their eligibility based on predetermined inclusion and exclusion criteria. The objectives, methodology, population, and significant findings (rate of cesarean section among breech deliveries in Ethiopia and prevalence of breech delivery) of the full-text studies in English were then reviewed further. The two authors came to a logical agreement to handle any questions that developed during the extraction process, and the final agreement was finalized with the assistance of the authors (AD and IM).

### 2.6 Data extraction

The two authors (IM and AE) extracted the data independently using a Microsoft Excel 2016 sheet after identifying articles that meet the inclusion criteria. Different variables of interest were considered in the extraction and presented in the table of contents. The accuracy of the data extraction was checked by comparing the results produced by the two authors. The information used for meta-analysis from the included articles was extracted, which included the total number of breech deliveries (N), frequency of the occurrence of cesarean sections (n), measure of associations with a 95% CI, and effect size.

### 2.7 Data item

The primary outcome of this meta-analysis is the rate of cesarean section among breech deliveries in Ethiopia. Breech delivery is the delivery of a fetus with a breech presentation. The secondary outcome of interest was the prevalence of breech deliveries. It is the proportion of breech deliveries among total deliveries in health institutions in Ethiopia.

### 2.8 The methodological quality of the studies

The risk of bias was assessed using the Newcastle-Ottawa scale, which is a validated tool for assessing the quality of non-randomized studies (cross-sectional studies) (20). The risk of bias assessment tool includes the following domains: selection domain, including representativeness of the sample, non-respondents, and ascertainment of the exposure (risk factor); comparability domain (the subjects in different outcome groups are comparable based on the study design or analysis, and confounding factors are controlled); and outcome domain, such as assessment of the outcome and statistical tests. The included studies’ methodological validity and the quality of their conclusions were scrutinized. Two authors (AD and IM) assessed and scored the quality of the study using NOS. The mean score of the authors was utilized to make the final decision. Based on their performance against each tool indicator, the included studies were categorized as high, moderate, or low quality. Good quality is defined as 80% or more, moderate quality as 60%–80%, and low quality as below 60%. The quality score of the eleven studies ranges from 6 to 9, with most studies (8 studies) scoring eight. All eleven studies were considered of adequate quality for inclusion in the analyses.

### 2.9 Statistical analysis

Statistical analysis was conducted using STATA 18 software. The meta-analysis data demonstrating adverse perinatal outcomes in breech deliveries among women in Ethiopia were presented using forest plots. A meta-analysis of the prevalence of adverse perinatal outcomes was done using a random effect model using the Der Simonian Liard method of analysis to reduce the heterogeneity of the included studies. Subgroup analyses were also performed based on several research parameters. Both bivariate and multivariate meta-regressions were performed to determine and identify the source of heterogeneity. A meta-analysis of observational studies was undertaken based on the recommendations of the *I*^2^ statistic given by Higgins et al. (an *I*^2^ of 75/100% and above implies considerable heterogeneity). To look for potential publication bias, the researchers utilized Egger's regression test, trim fill analysis, and a visual evaluation of a funnel plot. Sensitivity analysis was done using the leave-one-out meta-analysis method to identify the effect of a single study on the overall estimate and to identify the outliers.

## 3 Results

### 3.1 Search findings and risk of bias assessment

**Figure 1:**
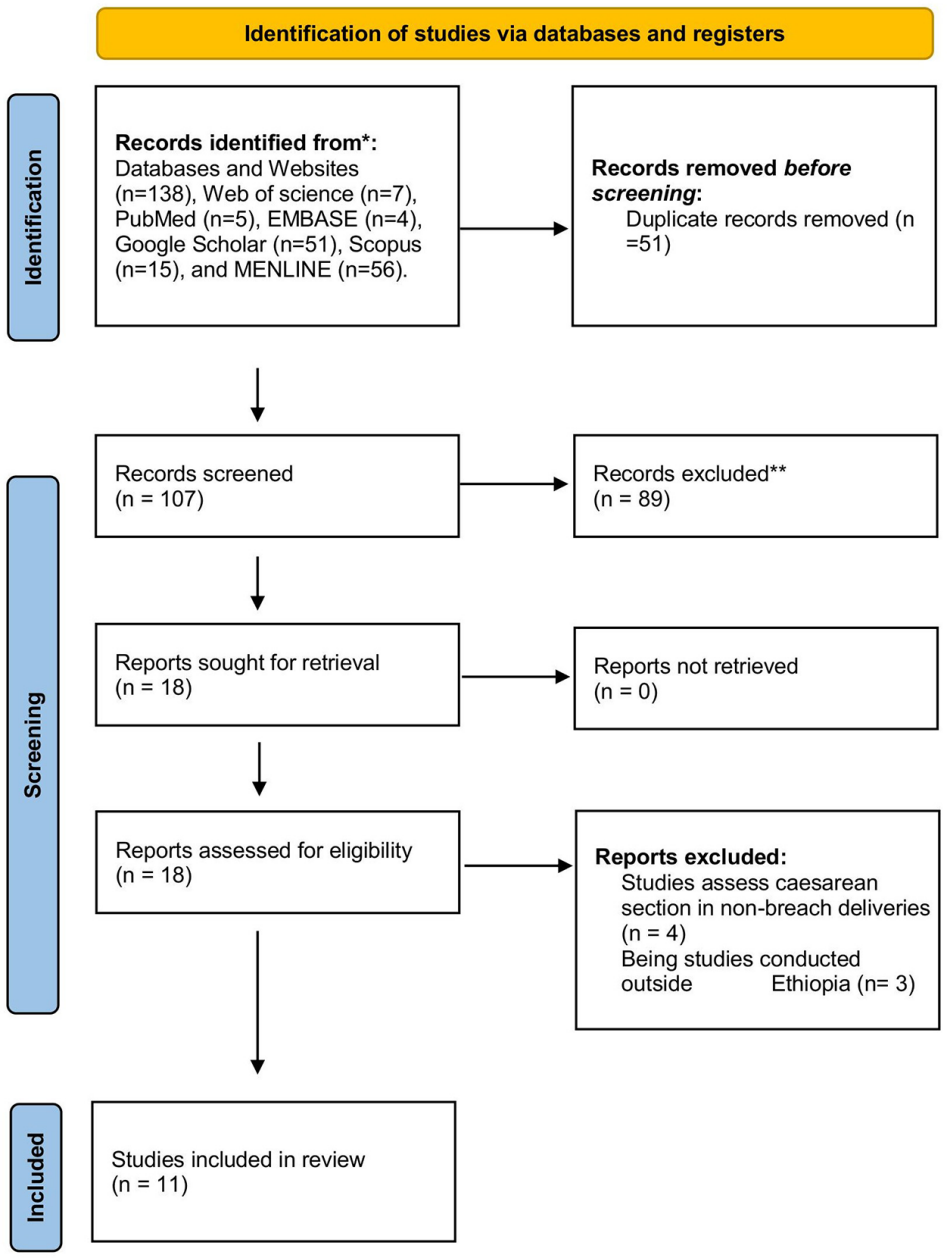
Flow chart showing the procedure of selecting studies for systematic reviews and meta-analysis up to 13/25/2023 G.C.

A total of 138 published and unpublished articles were found in databases and institutional repositories. Using Endnote and visual inspection, 31 publications were removed from all identified studies due to duplication. The remaining 107 studies were then maintained and screened based on title and abstract. After being vetted based on titles and abstracts, 89 were eliminated. Eighteen articles were considered eligible, and eight studies were removed because they assessed non-breech deliveries or were done outside Ethiopia. Finally, the systematic review and meta-analysis comprised 11 published and unpublished observational studies that met the inclusion criteria (Figure 1). A detailed review of the included research across eight domains yielded high-quality scores. Fortunately, all articles were included.

### 3.2 Characteristics of included studies

This systematic review and meta-analysis comprised eleven observational studies (10 cross-sectional and one case-control study) that assessed the prevalence of breech deliveries and the rate of cesarean section among breech deliveries in Ethiopia. The sample size of the included studies ranges from a minimum of 612 in a study by Wadajo AT (21) to a maximum of 11,546 in studies done by Fentahun et al. (22). The included articles were published between 1995 (23) and 2022 (24). Of all the studies, more than half (6) were conducted in the Oromia region (16–20), one study in Harari (16), one study in Amhara (17), one study in the southern nation and nationality region (18), and one study in Addis Ababa (19). A total of 57,236 mothers who gave breech deliveries at the health institution were included in this systematic review and meta-analysis. A summary of the main characteristics of the papers included in this systematic review and meta-analysis (Table 1).

**Table 1:** Characteristics of included studies in this systematic review and meta-analysis in 2023.

Studies	Setting	Design	Data collection method	Total delivery	total breech delivery	Vaginal delivery	Cesarean delivery	Prevalence of breech (%)
Debero Mere T. et al. (3)	Hospital	Cross sectional	Chart review	10,214	384	317	67	3.4
Assefa et al. (26)	Hospital	Cross sectional	Interview	2,029	108	42	66	5.3
Yared et al. (17)	Hospital	Cross sectional	Chart review	4,875	197	57	138	4
Fentahun et al. (22)	Hospital	Cross sectional	Chart review	11,546	374	235	139	3.3
Beyene et al. (28)	Hospital	Cross sectional	Chart review	5,628	164	98	66	3
Mekbib T.A., (19)	Hospital	Cross sectional	Chart review	7,170	291			4
Tilahun et al. (18)	Hospital	Cross sectional	Chart review	3,729	126	67	51	3.4
Dereje D. et al. (29)	Hospital	Cross sectional	Interview and chart review	3,388	227	111	116	6.7
Amano et al. (25)	Hospital	Cross sectional	Interview	4,480	208	163	45	4.6
Wadajo A.T., (21)	Hospital	Case control	Interview and chart review	612	153			25
Wabe M. (27)	Hospital	Cross sectional	Interview	3,565	106	66	40	3.1

### 3.3 Meta-analysis of the prevalence of breech deliveries

**Figure 2:**
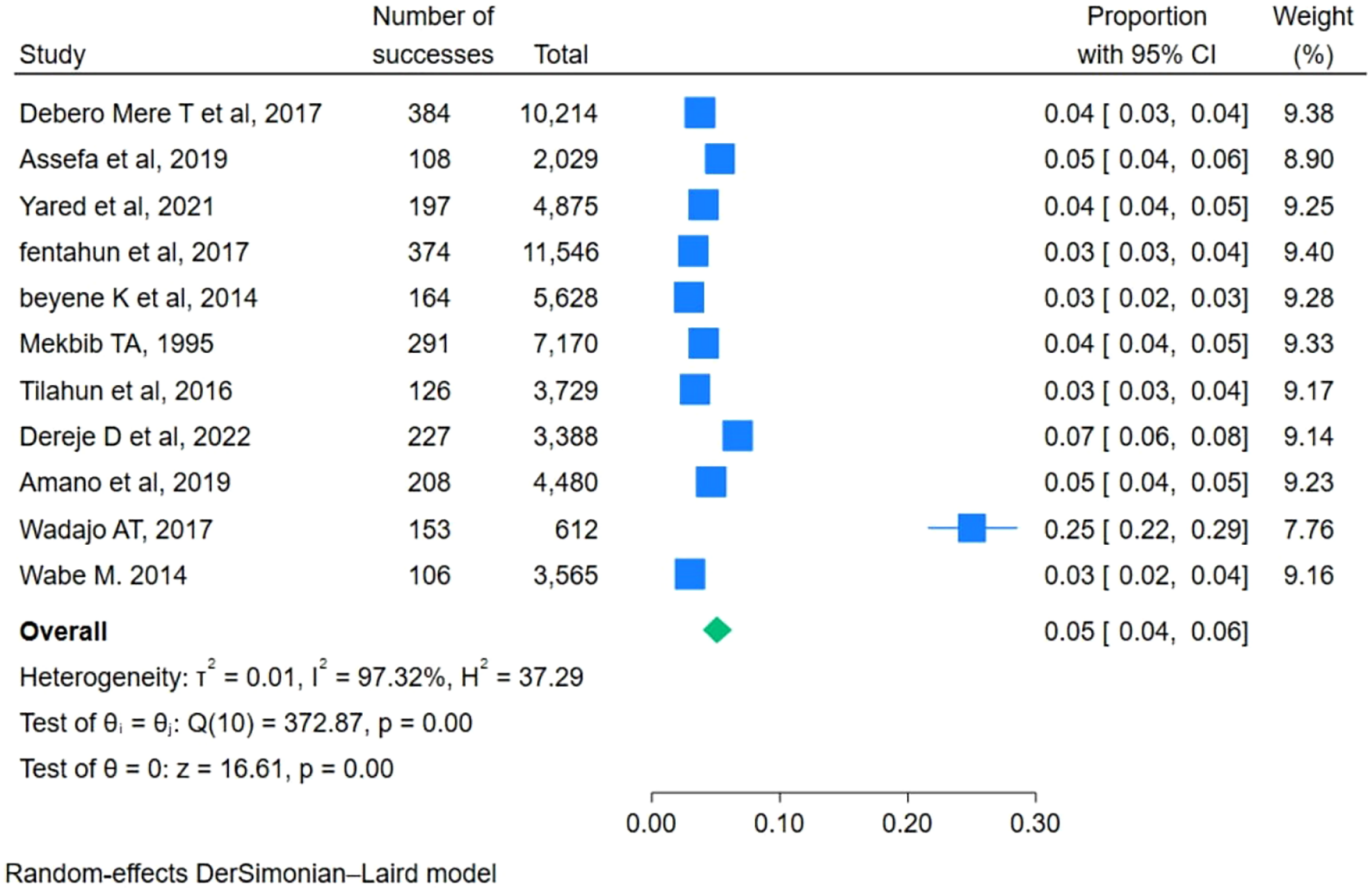
Pooled prevalence of breech deliveries among women in Ethiopica, 2023.

In this meta-analysis, a total of 11 studies were considered. The prevalence of breech delivery ranges from 3% (95% CI: 2–3) to a maximum of 25% (95% CI: 22–29). The pooled prevalence of breech deliveries among women in Ethiopia was 5% [95% CI: 4, 6]. There is significant heterogeneity among individual studies (*I*^2^ = 97.32%) (Figure 2).

### 3.4 Subgroup analysis of prevalence of breech deliveries

**Figure 3:**
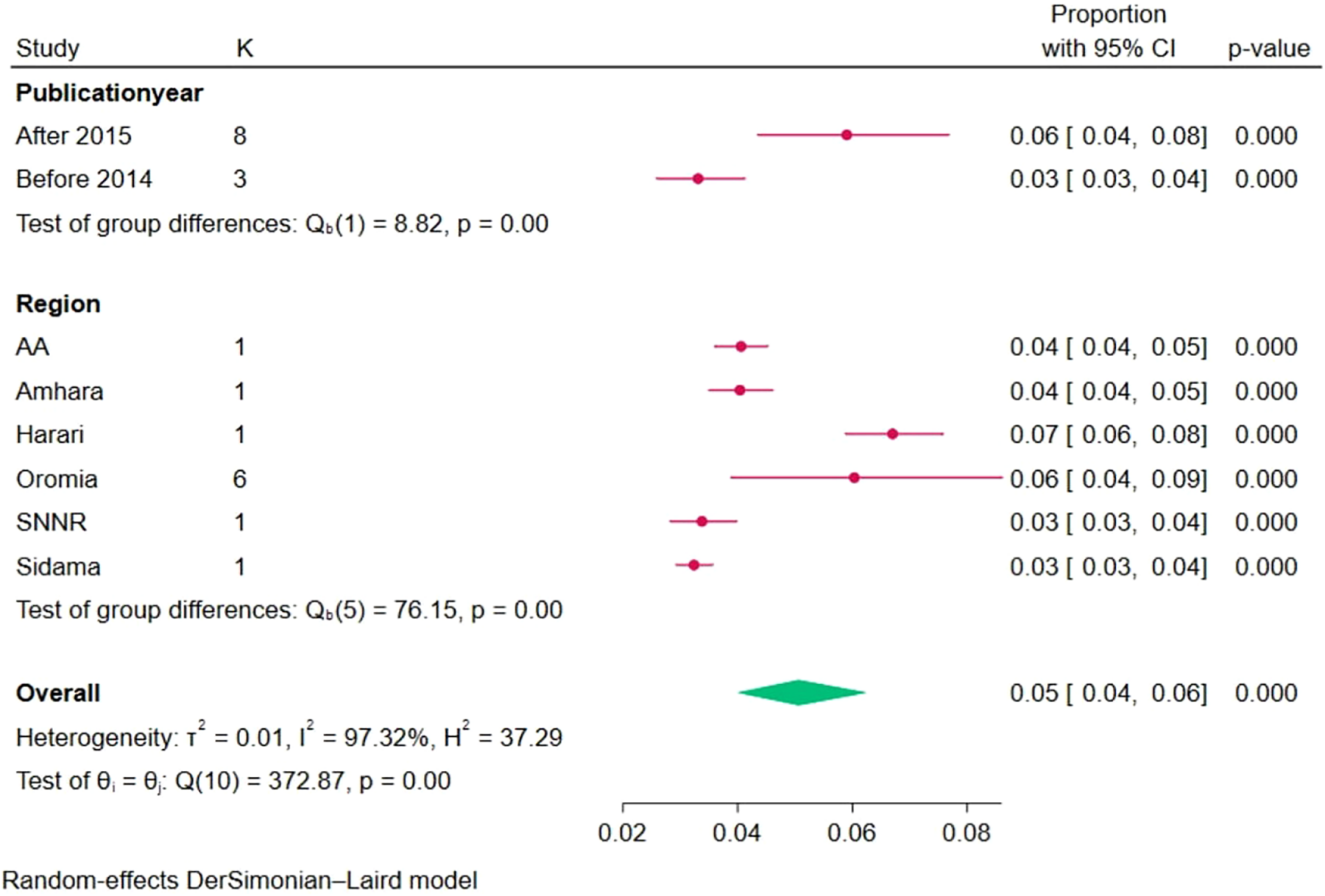
Subgroup analysis of the pooled prevalence of breech deliveries in Ethiopia based on region and publication year, 2023.

Based on subgroup analysis by publication year, the highest prevalence of breech deliveries among women in Ethiopia was observed among studies conducted after 2014, which was 6% (95% CI: 4–8). The heterogeneity among the subgroups was different, and there was a decline in heterogeneity in studies conducted during and before 2014. Based on subgroup analysis by region, the prevalence of breech deliveries among women in Ethiopia was observed among studies conducted in the Oromia region at 7% (95% CI: 6–8) (Figure 3).

### 3.5 Publication bias

**Figure 4:**
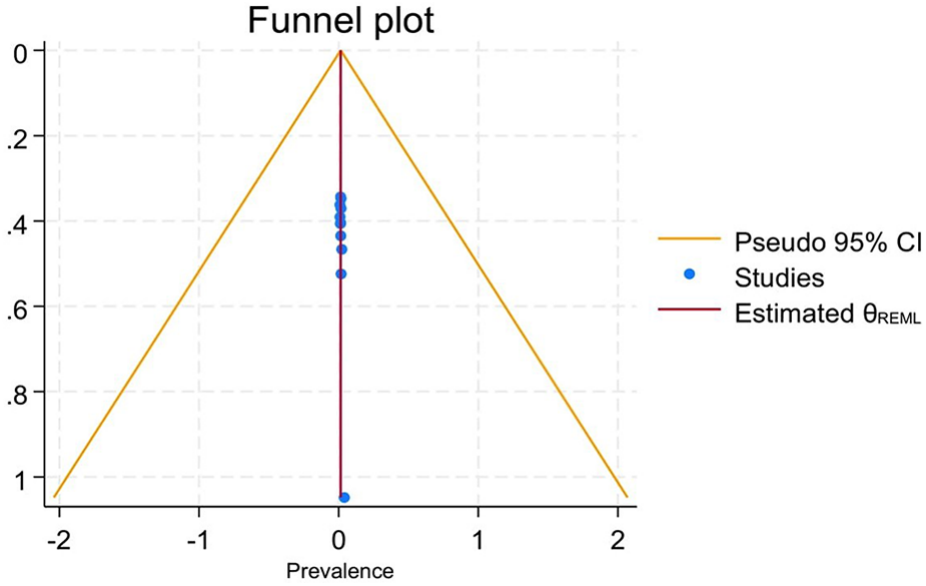
Funnel plot of the pooled prevalence of breech deliveries in Ethiopia, 2023.

To observe publication bias, a visual inspection of the funnel plot was carried out, and it shows that there was no publication bias observed despite significant heterogeneity among studies (Figure 4). Egger's test shows that there was no small study effect on the estimate (*P* = 0.87). Furthermore, trim fill analysis also showed no difference in observed or combinations of observed and imputed effect size estimates in the random effect model utilizing Der Simonian Liard.

### 3.6 Multivariate meta-regression

The pooled prevalence of breech deliveries among women in Ethiopia shows that there was heterogeneity and the I-square test statistics were significant; a meta-regression analysis was performed (*I*^2^ = 97.32, *P* value = 0.001). Both bivariate and multivariate meta-regressions were conducted using different variables. The meta-regression study discovered that sample size was the source of the heterogeneity. However, other study-level covariates were not statistically significant (Table 2).

**Table 2:** Bivariate and multivariate meta-regression analysis to check heterogeneity, 2023.

Variables	Coefficients	Standard error	*p*	95% CI
Total delivery^a^	−0.0000299	0.8306	0.001	−0.0000462, −0.0000136
Publication year^a^	0.0036934	0.0040067	0.357	−0.0041596, 0.0115465
Publication year^b^	0.0005868	0.0041603	0.888	−0.0075672, 0.0087409
Total delivery^b^	0.0004924	0.92806	0.001	−0.0000482, −0.0000119

^a^ Bivariate meta regression.

^b^ Multivariate meta regression.

### 3.7 meta-analysis of the rate of cesarean section among breech deliveries

**Figure 5:**
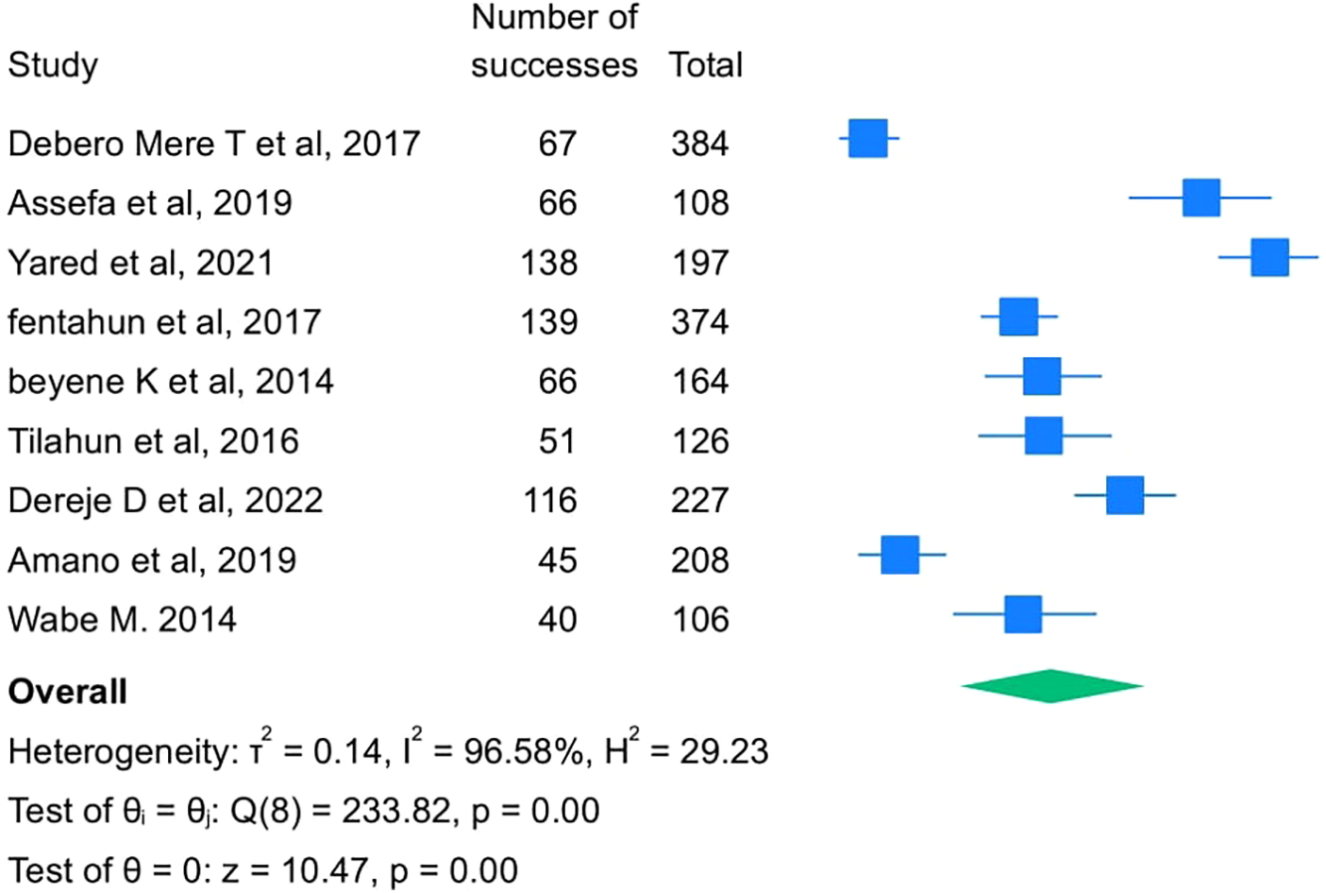
Rate of cesarean section among breech delivery among women in Ethiopica, 2023.

Of the eleven studies, nine report the rate of cesarean section among breech deliveries in Ethiopia (3, 17, 22, 25–29). Of them, the rate of cesarean section ranges from 17% (95% CI: 14–21) to 70% (95% CI: 63–76). The overall pooled cesarean sections rate among breech deliveries in Ethiopia was 41% (95% CI: 29–54) (Figure 5).

### 3.8 Subgroup analysis of the rate of cesarean section among breech delivery

**Figure 6:**
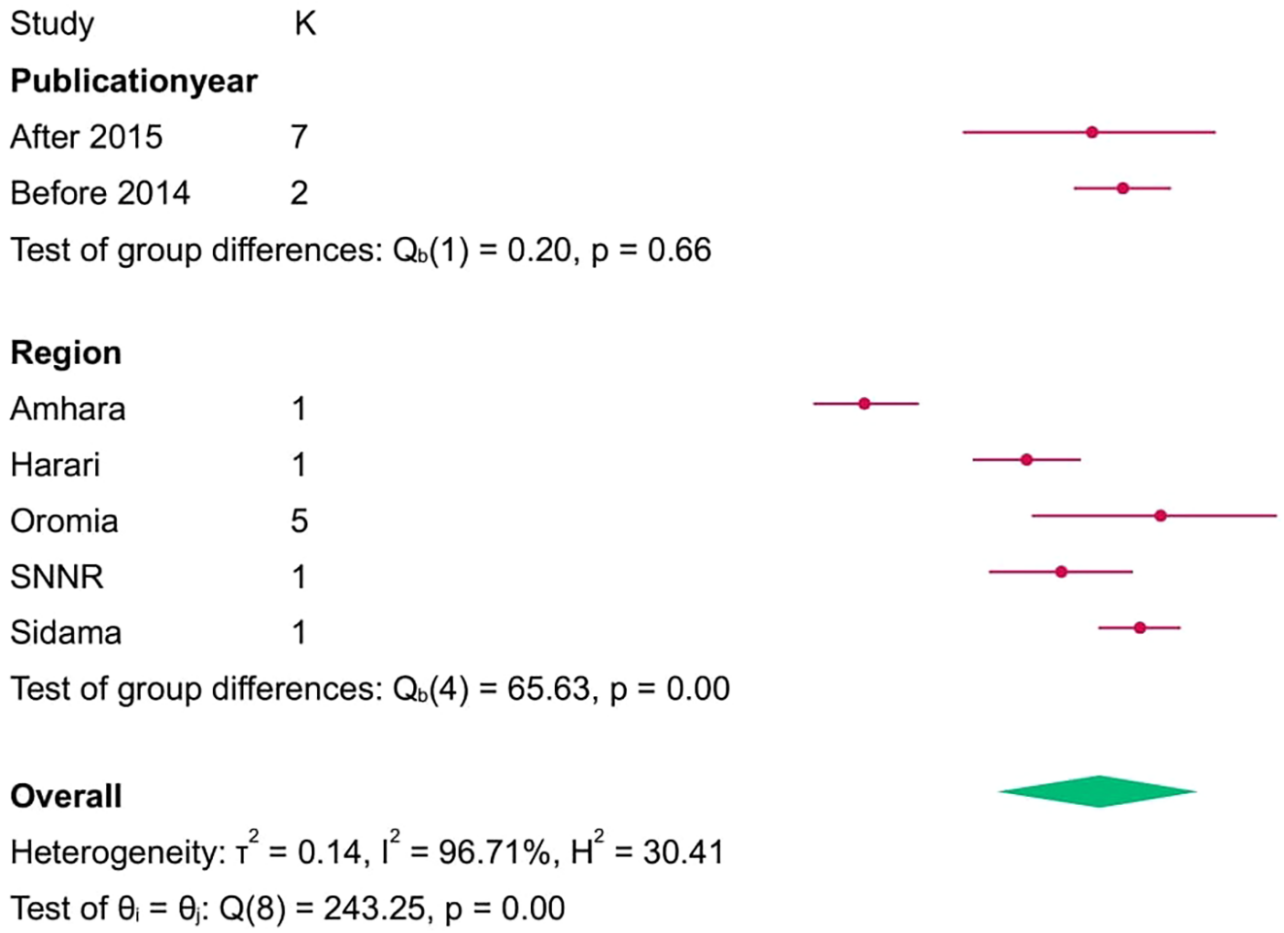
Subgroup analyis of the rate of cesarean section among breech delivery among women in ethipia, 2023.

Based on subgroup analysis by publication year, the highest rate of cesarean section in breech deliveries among women in Ethiopia was observed among studies conducted during and before 2014, which was 61% (95% CI: 55–67). Based on subgroup analysis by region, the highest rate of cesarean section in breech deliveries among women in Ethiopia was observed among studies conducted in the Oromia region, which was 65% (95% CI: 50–80) (Figure 6).

### 3.9 Sensitivity analysis

**Figure 7:**
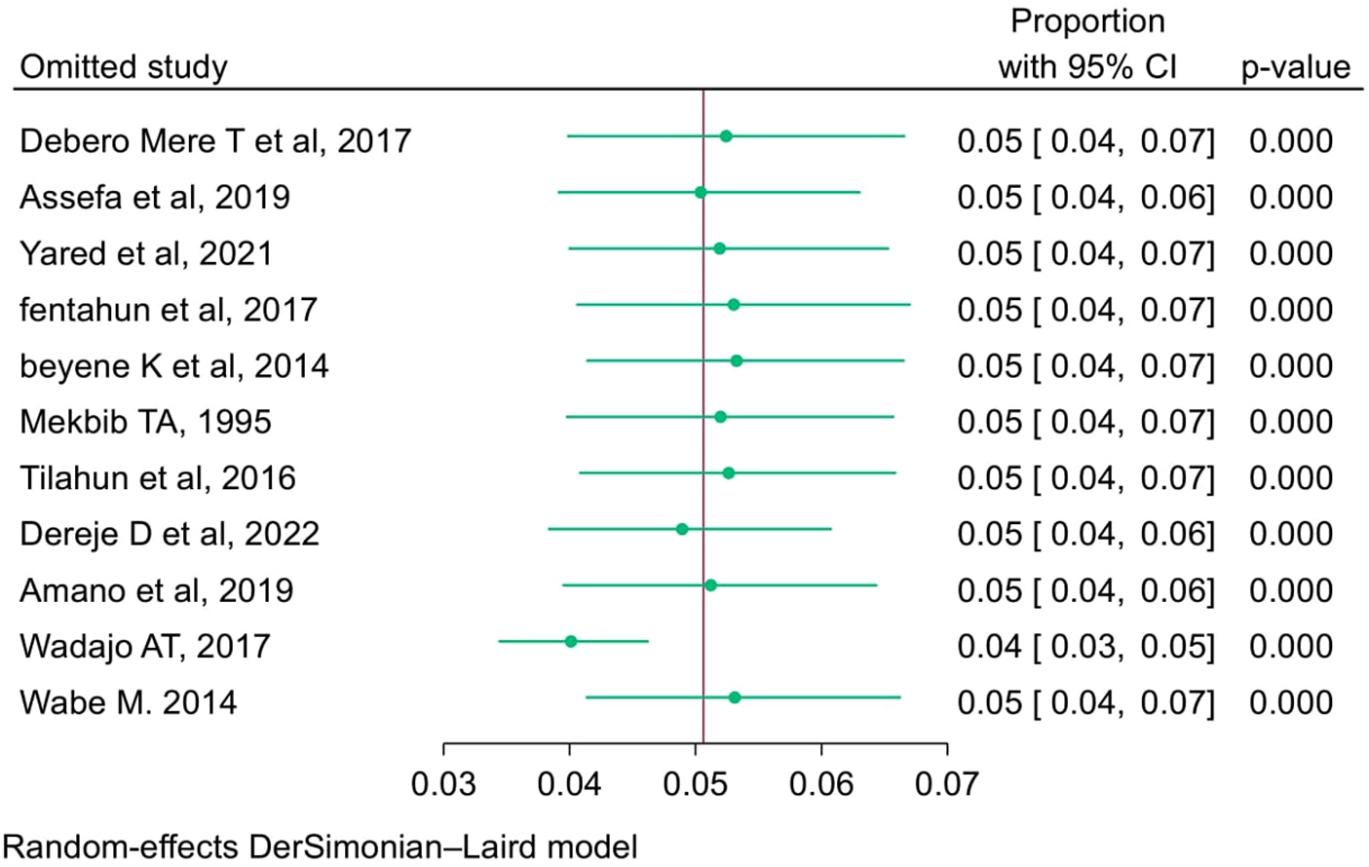
Leave one out meta-analysis of prevalence breech delivery among women in Ethiopica, 2023.

Leave one out of a meta-analysis. The Der Simonian Liard method was used to assess the effect of a single study on the overall estimate of the rest of the studies in the meta-analysis results and to identify outliers. As shown in the figure below, when individual studies were removed and the overall prevalence re-estimated, there was no such significant deviation from the previously determined prevalence (Figure 7). Furthermore, there were no outliers observed.

## 4 Discussion

The purpose of this systematic review and meta-analysis was to determine the rate of caesarean sections among women who gave breech deliveries in Ethiopia. In this review, the pooled prevalence of breech deliveries among women in Ethiopia was 5% [95% CI: 4, 6]. The overall pooled cesarean section rate among breech deliveries in Ethiopia was 41% (95% CI: 29–54). This finding pointed out that two out of every five pregnant women with breech presentation gave birth by cesarean section in Ethiopia. This is in line with the systematic review and meta-analysis conducted in Nigeria (30). This implies that a large portion of women with breech presentations end up with caesarian deliveries, which in turn predispose the women to various short-term and long-term surgical complications after caesarian sections (31, 32). Thus, as compared to vaginal delivery, pregnant women who gave birth through caesarian delivery were at higher risk for incisional hernia (33, 34), bowel obstruction (35), abdominal pain (36), and organ damage (32). The catastrophic sequels related to complications after caesarean sections are not only limited to the mother but also explicitly affect the neonates. Some of the prominent complications are birth asphyxia, trauma, jaundice, and hypoglycemia (37, 38). Hence, coordinated action and measures need to be taken to mitigate and alleviate potential risk factors that lead to caesarean sections.

According to this systematic review and met analysis, there is a significant difference in the years of publication, which explicitly confirmed the rate of caesarian section among the breech deliveries within the given years of publication. Thus, based on subgroup analysis by publication year, the highest rate of cesarean section in breech deliveries among women in Ethiopia was observed among studies conducted during and before 2014, which was 61% (95% CI: 55–67). Additionally, according to this systematic review and meta-analysis, the rate of caesarian section among breech deliveries showed a bit of a reduction after 2015, which was 57% (95% CI: 41, 72). This is related to the decline in breech delivery, which in turn led to reductions in cesarean sections in the given years.

Moreover, this systematic review and meta-analysis revealed that the rate of caesarean section among breech deliveries enormously varies across regions of the country. Accordingly, the highest rate was reported from the Oromia region, which was 65% (95% CI: 50, 80), whereas the lowest was reported from the Amhara region, accounting for 29% (95% CI: 23, 35). This variation could be attributed to the fact that a number of the original studies were included in reviews. For instance, the review entails five studies from Oromia and one study from the Amhara region. Hence, as ANC visits help in the early identification of breech presentation, they also greatly reduce the consequences of breech presentation, such as caesarian sections (39–41).

## 5 Strengths and limitations of the study

The study's strength is that the publications were found in a variety of genuine databases, websites, and institutional repositories. Another strength is that, to the best of the investigators’ knowledge, it is the first SRM on adverse perinatal outcomes in breech deliveries. However, the majority of the pieces came from a few regions. During the article search, only the English language was taken into account. Furthermore, unpublished studies were included in this review and may influence the overall estimate.

## 6 Conclusion

In this review, the pooled prevalence of breech deliveries among women in Ethiopia was 5%, and the overall rate of caesarian section among the breech deliveries was 41%. This finding pointed out that two out of every five pregnant women with breech presentation gave birth by cesarean section in Ethiopia. Therefore, the finding implies that both the government and all the concerned stakeholders shall be given particular emphasis made on strengthening antenatal care services and ensure more women have access to skilled healthcare professionals during childbirth. This can help in providing appropriate interventions, support to women and reducing the need for emergency and unnecessary CSs. The result of this research are a baseline data for future researchers to conduct further studies to better understand the reasons behind the high rates and identify potential interventions and solutions specific to the African context.

## Data Availability

The original contributions presented in the study are included in the article/Supplementary Material, further inquiries can be directed to the corresponding author.
